# Flow Cytometric Analysis of the Cytotoxic T-Cell Recall Response to *Theileria parva* in Cattle Following Vaccination by the Infection and Treatment Method

**DOI:** 10.3390/vetsci8060114

**Published:** 2021-06-18

**Authors:** Mahmoud M. Elnaggar, Donald P. Knowles, William C. Davis, Lindsay M. Fry

**Affiliations:** 1 Department of Veterinary Microbiology & Pathology, College of Veterinary Medicine, Washington State University, Pullman, WA 99164, USA; Mahmoud.elnaggar@alexu.edu.eg (M.M.E.); dknowles@wsu.edu (D.P.K.); davisw@wsu.edu (W.C.D.); 2 Department of Microbiology, Faculty of Veterinary Medicine, Alexandria University, Alexandria 22758, Egypt; 3 Animal Disease Research Unit, USDA-ARS, Pullman, WA 99164, USA

**Keywords:** flow cytometry, East Coast fever

## Abstract

The apicomplexan hemoparasite, *Theileria parva*, causes East Coast fever (ECF), a frequently fatal disease of African cattle. Vaccine development has been impeded by incomplete understanding of protective immunity following natural exposure or the infection and treatment method (ITM) of immunization. This is attributable to a paucity of methods to characterize the memory T-cell repertoire following infection. To overcome this impediment, assays developed to study the immune response to other intracellular pathogens were adapted for use in studies with *T. parva* to enable definition of the phenotype and function of effector T cells in *T. parva*-immune cattle, facilitating vaccine development. As reported herein, stimulation of peripheral blood mononuclear cells (PBMC) from ITM-immunized steers with irradiated, autologous, *T. parva*-infected cell lines elicited a proliferative recall response comprised of CD45R0^+^/CCR7^−^ CD4^+^ and CD8^+^ T cells. Subsequent co-incubation of stimulated cultures with infected cells demonstrated the presence of cytotoxic T cells (CTLs) with the ability to kill infected cells. Comparison of CTL activity in cultures depleted of CD4^+^ or CD8^+^ T cells demonstrated CTL activity was primarily attributed to CD8^+^ T cells. Importantly, stimulation of PBMC from vaccinated steers always elicited proliferation of CD4^+^ and CD8^+^ T cells. This was the first important observation obtained from the use of the assay described herein.

## 1. Introduction

The tick-borne, apicomplexan parasite *Theileria parva* (*T. parva*) is the causative agent of East Coast fever (ECF). ECF kills over a million cattle annually in sub-Saharan Africa (reviewed in [1]). Mortality rates are highest in *Bos taurus* cattle breeds, and in *Bos indicus* breeds raised in non-endemic regions. Most losses are incurred by pastoralist farmers. As a consequence, ECF-induced livestock morbidity and mortality remains a leading cause of poverty in the region.

Once transmitted to cattle or buffalo by a *Rhipicephalus appendiculatus* tick, *T. parva* sporozoites rapidly enter B and T lymphocytes, and develop to multinucleated schizonts [2]. The schizont lies free within the host cell cytoplasm and induces neoplasia-like transformation that immortalizes the host lymphocyte [3]. The parasite divides in concert with the transformed lymphoblast during schizogony, and it is this life stage that induces ECF in cattle. During acute ECF, the lymph nodes increase in size as they become infiltrated with *T. parva*-infected cells and blasting lymphocytes [4]. Animals develop severe leukopenia, protracted pyrexia, and anorexia. Severely affected cattle exhibit pronounced phenotypic changes in monocyte populations [5], and eventually develop respiratory failure. Recent histopathology studies in lethally infected cattle revealed that terminal pulmonary changes are characterized by systemic macrophage activation, vessel infiltration, and vasculitis, rather than lymphoproliferation alone [6]. Indeed, alterations in the innate immune response are correlates of severe disease in ECF-infected cattle [7]. Although it is likely that transformed lymphocytes play a role in macrophage infiltration and activation, the mechanisms involved remain to be elucidated.

The use of the infection and treatment method (ITM) of immunization, in which cattle are inoculated with ground, *T. parva*-infected ticks and co-treated with oxytetracycline, is quite effective at protecting cattle from similar strains, but can fail when cattle are exposed to heterologous strains of the parasite [8]. In addition, ITM stabilates are expensive to manufacture, and require liquid nitrogen storage and antibiotics [9,10]. Nevertheless, the ITM has shown that development of protective immunity is possible, and that protection can be broadened by use of combinations of *T. parva* isolates with different antigenic signatures (reviewed in [1]). The availability of immune cattle provided opportunity to begin analysis of the immune response and the factors associated with development of protective immunity. Use of adoptive transfer of CD8^+^ T cells from a calf vaccinated by the ITM to a genetically identical twin unvaccinated calf revealed complete protection is mediated by CD8^+^ cytotoxic T cells (CTLs) [11]. Efforts to replicate this level of protection using individual antigenic proteins expressed by schizonts, identified as targets of the immune response, have yielded some promising results and many challenges [12,13,14,15].

Important insights into the immune response to *T. parva* were gained from these studies. However, the Ag-specific CTLs elicited by immunization or ITM were used up in one-dimensional assays, limiting opportunities for further characterization of the protective CTL response, a limitation also encountered in previous studies [13]. Apart from CD8^+^ T-cell killing of *T. parva*-infected lymphocytes, there are no other known immune correlates of protection in *T. parva*. Indeed, other common in vitro measures of T-cell responsiveness, including the production of interferon gamma and cellular proliferation, provide no indication of protection from subsequent challenge. This paucity of information significantly hinders vaccine development, as the complete profile of a successful immune response remains nebulous.

Concurrent work with porcine reproductive and respiratory syndrome virus (PRRSV) in pigs and *Mycobacterium* spp. in cattle suggests these limitations may be overcome by adapting flow-cytometry-based cytotoxicity assays to the *T. parva* system [16,17]. These cytotoxicity assays were originally developed to study killing of PRRSV or *Mycobacterium* spp.-infected macrophage target cells by CTLs, without loss of the CTLs, thereby allowing more complete characterization of the protective CTL response. The objective of the present study was to adapt these methods for use in characterizing CTLs that develop in cattle following *T. parva* ITM immunization.

## 2. Materials and Methods

### 2.1. Animals and Cell Lines

Four Holstein steers were initially infected with *T. parva* using the infection and treatment method (ITM), as previously described [6,7,18,19]. In brief, steers were inoculated subcutaneously with a lethal dose (1 mL comprised of the product of ten, macerated pairs of infected tick salivary glands) of *T. parva* Muguga sporozoite stabilate (Muguga genotype only, not the Muguga cocktail) in the left parotid region, and long-acting oxytetracycline (20mg/kg) was administered intramuscularly at the same time. Steers were clinically managed as previously described [7]. At intervals of 6–20 months thereafter, steers were boosted via subcutaneous injection of the same dose of *T. parva* Muguga sporozoite stabilate, at which point, they developed no clinical signs due to *T. parva* immunity. Blood was collected from infected steers during the 3–12-week post-challenge interval to analyze the T-cell proliferative response and to generate *T. parva*-specific CTL lines for use in the development of the FC-based cytotoxicity assay. Experiments were approved by the Washington State University Institutional Animal Care and Use Committee (ASAFs 4980 and 6622).

*T. parva*-infected lymphocyte lines were established for each steer by in vitro infection of PBMC using the Muguga sporozoite stabilate as previously described [7,15,20,21]. PBMC were isolated from whole blood using density centrifugation with Histopaque (Sigma-Aldrich, St. Louis, MO, USA). The success of infection was verified via light microscopic evaluation of Giemsa-stained cytospin smears for *T. parva* schizonts 14–21 days post-incubation of PBMC with *T. parva* sporozoites. Once infection was verified, infected cell lines were maintained by sub-culturing 1:2 or 1:3 with fresh medium every 2–4 days as needed, as indicated by medium color change from red to yellow, and cells forming confluent, non-adherent sheets or clumps. Unless treated with theileriacidal compounds, *T. parva*-infected lymphocyte lines behave as immortal lines, and are often cultured for years [22]. All cell cultures were conducted in complete RPMI (cRPMI): RPMI 1640 (Gibco, Gaithersburg, MD, USA) supplemented with 10% calf bovine serum, 20 mM HEPES buffer (Gibco), 50 µM β-mercaptoethanol (Gibco), 2 mM L-glutamine (Gibco), and 50 µg/mL gentamicin (Gibco), and were maintained at 37 °C/5% CO_2_. All monoclonal antibodies (mAbs) used in this study are listed in Table 1.

### 2.2. Generation of T. parva-Specific Effector T Cells

*T. parva*-specific effector T cells were generated as previously described [22], with slight modifications. Blood from *T. parva*-immune animals was collected in anticoagulant citrate dextrose (ACD). PBMC were isolated by density gradient centrifugation using Histopaque (density 1.077 g/mL; Sigma-Aldrich, St. Louis, MO USA). PBMC from immune steers were cultured in cRPMI and stimulated with autologous, γ-irradiated (50 Gy) *T. parva*-infected cell lines at a ratio 20:1 for 6 days in cRPMI at 37 °C/5% CO_2_. After the first stimulation, cells were collected and subjected to density gradient centrifugation to remove dead cells. Harvested cells were phenotyped and depleted of NK and γδ T cells using anti-NK and -γδ T-cell monoclonal mAbs (Table 1) (1 µg/10^6^ cells for each mAb) using rat anti-mouse IgG2a+b magnetic microbeads (Miltenyi Biotec, Waltham, MA, USA) according to manufacturer protocols. The depleted cells, comprised of CD4^+^ and CD8^+^ T cells, were resuspended in cRPMI and subjected to a second 5–6 days round of stimulation with autologous irradiated infected cell lines. After the second stimulation, cells were collected and again subjected to density gradient centrifugation to remove dead cells. Harvested cells were phenotyped and used to conduct the CTL assay and to generate subcultures of CD4^+^ and CD8^+^ T cells. CD8^+^ T cells were positively selected using a CD8-specific mAb (Table 1) (1 µg/10^6^ cells) and rat-anti-mouse IgG2a+b magnetic microbeads as previously described. The separated CD8^+^ T cells were phenotyped to verify purity and the residual preparations of CD4^+^ T cells were also phenotyped for purity. Preparations of CD4^+^ and CD8^+^ T cells were then expanded in cRPMI containing bovine IL-2 (10 ng/mL, Kingfisher, USA) and used in a cytotoxicity assay.

Combinations of mAbs specific for CD4, CD8, γδ T cells, CD45R0 (expressed on memory T cells), CCR7, and CD25 were used to determine the composition of lymphocyte cultures after each round of stimulation, after depletion of NK and γδ T cells, after positive selection of CD8^+^ T cells, and before setting up the cytotoxicity assay co-culture. Staining of cells was done as previously described using fluorochrome-conjugated goat anti-mouse isotype-specific second step antibodies [24]. Data were collected with a Becton Dickinson FACSCalibur flow cytometer and Cell Quest software (BD Immunocytometry Systems San Jose, CA) were used to collect the data. De Novo FCS 6 software was used to analyze the data (DeNovo Software, Pasadena, CA, USA).

### 2.3. Flow Cytometric Cytotoxicity Assay

A three-color flow cytometric assay was used to monitor killing of autologous, *T. parva*-infected target cells by unseparated parent cell lines and by separated, *T. parva*-specific, CD8^+^ T and CD4^+^ T-cell lines. Target cells were prepared just before use. Infected cells were harvested from culture, subjected to density gradient centrifugation to remove dead cells, and resuspended at 2 × 10^6^ cells/mL in RPMI with CellTracker Deep Red (Thermo-Fisher, USA) at a 1:1000 dilution for 30 min at 37 °C. Cells were then washed and resuspended in cRPMI. Unseparated preparations of *T. parva*-specific CD4^+^ and CD8^+^ T cells were subjected to density gradient centrifugation to remove dead cells and resuspended in cRPMI. Following separation and further culture in IL-2, preparations of separated CD4^+^ and CD8^+^ T cells were cleared of dead cells using the same procedure.

Effector cells were co-cultured with labeled, autologous infected cells for 6, 24, and 48 h at an effector:target (E:T) cell ratio of 40:1 (for the parent and CD4^+^ T-cell lines) and 20:1 (for the CD8^+^ T-cell lines). One well containing only labeled target cells was included as a control to assess spontaneous target cell death. At each time point, one well of each cell preparation was collected, pelleted, resuspended in annexin V binding buffer (BD Bioscience, USA), and mixed with annexin V PE and 7-AAD (20 µL each). Cell preparations were then incubated for 15 min at room temperature. Labeled cells were analyzed by flow cytometry within one hour of staining. At least 3 × 10^4^ target cells were collected from each sample by gating on the CellTracker Deep Red-labeled cells.

### 2.4. Data Analysis

FCS Express software (De Novo software, Los Angeles, CA, USA) was used to analyze all FC data. Statistical analysis of data was performed using GraphPad Prism (GraphPad Software, San Diego, CA, USA). A one-tailed Student’s *t*-test was used to analyze the difference between different treatments. *p*-values < 0.05 were considered significant.

## 3. Results

### 3.1. Recall Response to Stimulation of PBMC from T. parva-Immune Steers with Autologous T. parva-Infected Lymphocytes

**Figure 1:**
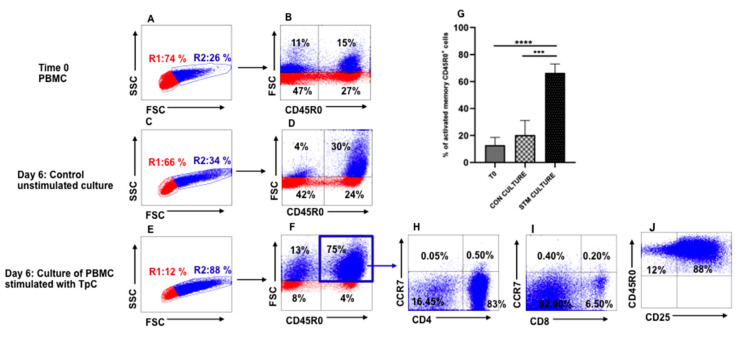
Analysis of the proliferative T-cell response to stimulation with autologous, irradiated, *T. parva*-infected cell lines (infected cell lines). Illustration of selective gating and the color coding strategy used to identify and track proliferation and expansion of antigen-specific memory T cells based on size increase of cells undergoing blastogenesis. Gate R1 distinguishes small, non-proliferating, resting lymphocytes and gate R2 distinguishes proliferating cells of increased size. An additional gate was used to isolate and quantify the frequency of antigen-specific memory T cells proliferating in response to stimulation with infected cell lines, displayed in FSC vs. CD45R0. Representative profiles showing the proportion of large memory cells present in PBMC at the initiation of culture (**A**,**B**) with PBMC cultured for six days without infected cell line stimulation (**C**,**D**), and cultured for six days with infected cell line stimulation (**E**,**F**) revealed an increase in antigen-specific memory T cells by six days of stimulation with infected cells. (**G**) Summary of the recall memory T-cell response of all four immunized steers to stimulation with autologous, irradiated infected cell lines. (**H**,**I**) Representative profiles showing the phenotype and frequency of memory CD4^+^ (**H**) and CD8^+^ (**I**) T cells proliferating in response to stimulation with infected cells lines. (**J**) Representative profile showing most of the antigen-specific proliferating memory T cells express CD25 by six days of stimulation with infected cell lines. CON CULTURE, unstimulated control culture; STM CULTURE, stimulated cultures. A one-tailed Student’s *t*-test was used to compare memory T cells present in unstimulated cultures at the initiation of culture (T0) with memory T cells present in unstimulated (*p* < 0.0001, ***) and six-day infected-cell-line-stimulated cultures (*p* = 0.0003, ****).

A series of experiments was conducted with PBMC from steers immunized using the ITM to develop a flow cytometric assay to characterize the CTL recall response following stimulation of PBMC with autologous, irradiated *T. parva*-infected cells. As described, the final cell preparations used in the assays were cleared of dead cells and phenotyped to verify that they were comprised of CD4^+^ and CD8^+^ T cells or contained only CD4^+^ or CD8^+^ T cells. As illustrated in Figure 1, electronic gates and color coding were used on PBMC displayed in side light scatter (SSC) vs. forward light scatter (FSC) to detect changes in cell size associated with cell proliferation. In this format, gate R1 distinguishes small, non-proliferating, resting lymphocytes and gate R2 distinguishes proliferating cells of increased size associated with blastogenesis [20,21]. An additional gate was used to isolate and quantify the frequency of antigen-specific memory T cells proliferating in response to stimulation with autologous, irradiated, infected cell lines, displayed in FSC vs. CD45R0. Comparison of a set of representative profiles showing the proportion of large memory cells present in PBMC at the initiation of culture (Figure 1A,B) with PBMC cultured for six days without infected cell line stimulation (Figure 1C,D), and cultured for six days with infected cell stimulation (Figure 1E,F) revealed an increase in antigen-specific memory T cells by six days of stimulation with infected cell lines. Repetition of the assay using PBMC from all four immunized steers showed the proliferative response to stimulation with infected cell lines was consistent (Figure 1G).

Further phenotypic analysis of the antigen-specific memory T cells showed that the majority of the cells were CD4^+^ T cells, and that both CD4^+^ and CD8^+^ T cells were CCR7 (CD197) negative, consistent with the phenotype of effector memory T cells (Figure 1H,I). Analysis of expression of CD25, expressed on activated cells, showed that the majority of antigen-specific memory T cells expressed the activation marker, CD25 (Figure 1J).

### 3.2. Flow Cytometric Detection of T-Cell Mediated Killing of Autologous, T. parva-Infected Cells

**Figure 2:**
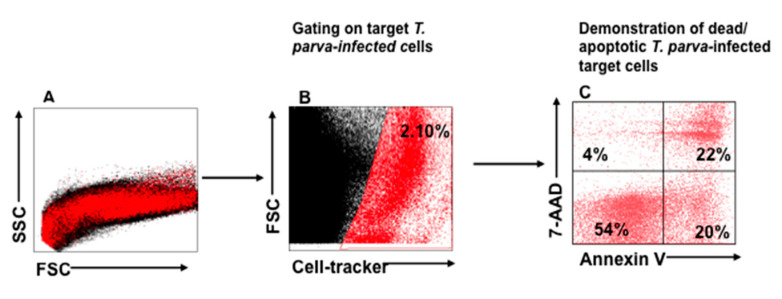
Gating strategy used to isolate labeled *T. parva*-infected target cells for analysis of CTL-mediated killing. (**A**) Representative mixture of CTLs and autologous, irradiated *T. parva*-infected cells (target cells) labeled with CellTracker Deep Red, annexin V, and 7-AAD after co-incubation for 6 h, displayed in SSC vs. FSC. (**B**) Selective gate used to isolate labeled cells for analysis displayed in FSC vs. CellTracker. (**C**) Gated infected cells displayed in 7 AAD vs. annexin V to show the proportion of infected cells undergoing apoptosis. The cells in the lower left quadrant show the proportion of live target cells remaining after incubation with CTLs for 6 h. The lower right quadrant shows the proportion of cells undergoing the initial stages of apoptosis (annexin V+/7AAD−). The upper right quadrant shows the proportion of target cells in late stages of apoptosis (annexin V+/7AAD+), and the upper left quadrant shows the proportion of target cells undergoing necrosis/non-apoptotic mechanisms of death (annexin V−/7AAD+).

A flow cytometric cytotoxicity assay was developed to directly assess killing of infected cells by CTL lines generated from PBMC of immunized steers. For this assay, a method was developed to distinguish infected cells, as target cells, from effector CTLs. The method was modeled after those used to study killing of virus-infected cells by CTLs, in which virus-infected target cells are labeled with a fluorescent viability dye [16,25]. In our assay, CellTracker Deep Red viability dye was used to label infected target cells. The annexin V PE and 7-AAD assay kit was then used to monitor CTL-mediated killing of infected target cells, as assessed by target cell apoptosis and cell death following the lethal hit mediated by the perforin-granzyme B pathway (Figure 2). In this format, visualized cells are positive for annexin V during early apoptosis and are also positive for 7-AAD during late apoptosis/cell death (early apoptosis, annexin V+/7-AAD-; late apoptosis, annexin V+/7AAD+; necrotic cells, annexin V−/7AAD+).

**Figure 3:**
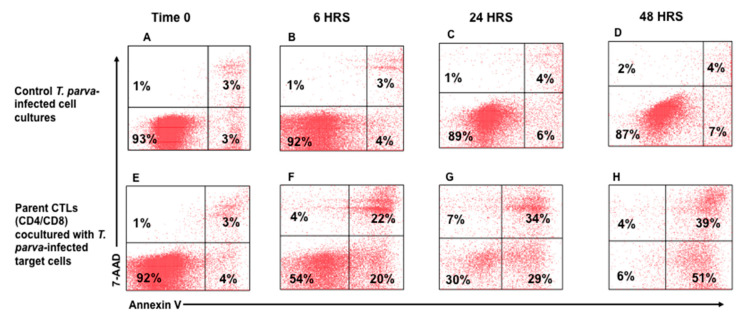
Detection of apoptotic and dead *T. parva*-infected target cells in preparations incubated with *T. parva*-specific CD4^+^ and CD8^+^ T cells. Representative preparation of infected target cells incubated alone (**A**–**D**) and with *T. parva*-specific stimulated CD4^+^ and CD8^+^ T-cell cultures (**E**–**H**) for 0, 6, 24, and 48 h. These results demonstrate that the proportion of apoptotic and dead infected target cells increases over time when incubated with *T. parva*-specific effector T cells. The lower left quadrants highlighted in green show the relative temporal changes in the proportion of viable target cells remaining at different time points following incubation with effector T cells. Little or no change in cell viability was evident in the culture of target cells maintained concurrent with the time target cells were co-cultured with parent cultures of effector cells containing CD4 and CD8 T-cell subsets. The results show there was a time dependent reduction in viable target cells. This was related to the time taken for the CTLs to deliver their lethal ‘kiss of death’ and move on to the next target cell.

**Figure 4:**
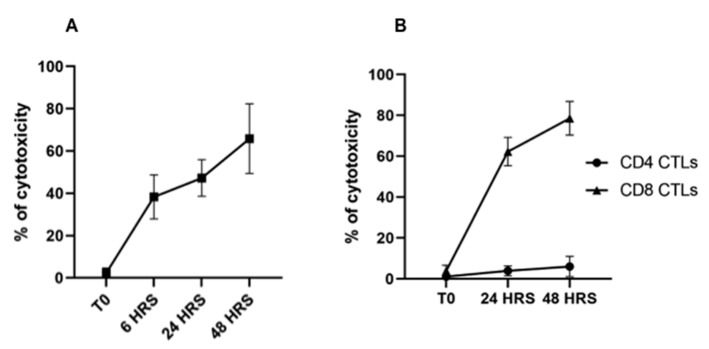
Comparison of the proportion of apoptotic and dead *T. parva*-infected target cells present in preparations incubated with *T. parva*-specific CD4^+^ and CD8^+^ T cells. (**A**) Display of dead and apoptotic infected target cells detected at 0, 6, 24, and 48 h incubation with CD4^+^ and CD8^+^ T cell parent cultures from *T. parva*-immunized steers stimulated ex vivo with autologous, irradiated, *T. parva*-infected cell lines. (**B**) Display of dead and apoptotic infected target cells detected at 0, 24, and 48 h incubation with CD4^+^ T cells (circles) or CD8^+^ T cells (triangles) previously stimulated ex vivo with irradiated, autologous *T. parva*-infected cell lines. Although most cytotoxic activity was mediated by CD8^+^ T cells, CD4^+^ T-cells also exhibited significant levels of infected target cell killing.

As described above, two rounds of stimulation were used to generate CTLs for the assay. CTL-mediated killing of infected cells was assessed after 6, 24, and 48 hrs. of co-culture with autologous, irradiated infected cell lines. An infected cell-only culture was included as a control to monitor the proportion of infected cells undergoing spontaneous apoptosis in the same timeframe. At the time of analysis, a selective gate was placed on the CellTracker Deep Red-labeled target cells to exclude CTLs from analysis (Figure 2A,B), followed by display of 7-AAD and annexin V on infected target cells to show the proportion of early and late apoptotic cells present in the preparation (Figure 2C). Although there was some loss of target cells attributable to CTL-mediated lysis, comparison of the CTL response in unseparated CD4^+^ and CD8^+^ T-cell cultures revealed the proportion of infected cells undergoing apoptosis increased substantially over time, from 3% at time 0 (Figure 3E), to 39% at 48 h (Figure 3F–H), especially when compared to infected target cells cultured without effector T cells (Figure 3A–D). The percent of early apoptotic and late apoptotic (dead) cells was considered the sum of 7-AAD^−^/annexin V^+^ and 7-AAD^+^/annexin V^+^ labeled, infected cells. The percentage of cytotoxicity (specific cytotoxic activity) was calculated using the following equation: % specific cell death = (% of dead infected cells—% of spontaneous dead infected cells)/(100% of spontaneous dead infected cells) × 100, as previously described [26]. Analysis of data obtained from comparison of CTL activity from all four immunized steers revealed a significant increase in the mean percentages of cytotoxicity mediated by parent unseparated cultures of CD4^+^ and CD8^+^ T cells beginning at 6 h (38.29% ± 10.45), 24 h (47.21% ± 8.69), and 48 h (65.81% ± 16.46) compared to T0 (2.34% ± 2.36) (Figure 4A).

**Figure 5:**
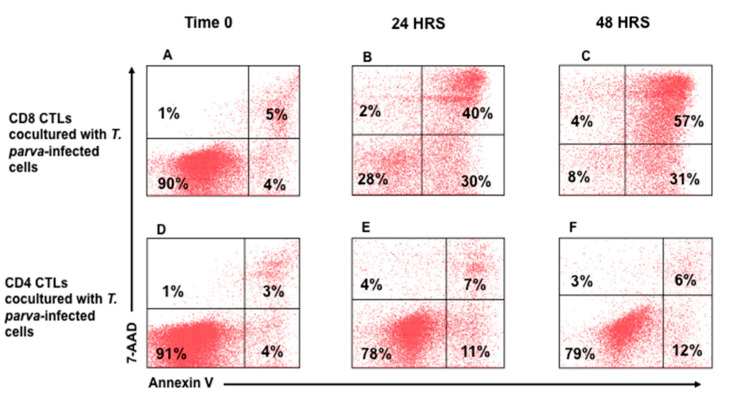
Flow cytometric comparison of CTL activity mediated by *T. parva*-specific CD4^+^ and CD8^+^ T cells. Representative flow cytometric profiles showing the proportion of apoptotic and dead *T. parva*-infected target cells present in preparations incubated for 0, 24, or 48 h with *T. parva*-specific, CD8^+^ (**A**–**C**) or CD4^+^ (**D**–**F**) T cells. Comparison of the lower left quadrants of the target cells incubated with CD4 or CD8 T cells show a clear reduction in viable target cells in the presence of separated CD8 T cells. This documents that the death of the target cells is attributable to a lethal hit mediated by CD8 T cells.

In further studies, parent cultures of CTLs were separated into preparations containing CD4^+^ or CD8^+^ CTLs alone. The cultures were expanded in the presence of bovine IL-2 before use. Two time points of 24 h and 48 h were used to compare the level of killing mediated by cultures of CD4^+^ or CD8^+^ T cells. Comparison of CTL activity demonstrated killing of infected target cells was mediated predominantly by CD8^+^ T cells in a time-dependent manner (Figure 4B and Figure 5A–C), with an average of cytotoxicity of 62.25 (±6.96) and 78.57 (±8.21) at 24 and 48 hrs., respectively. CD4^+^ CTLs showed minimal cytotoxic activity of 3.96% (±2.38) and 6.06% (±4.95) at 24 and 48 h, respectively, (Figure 4B and Figure 5D–F).

## 4. Discussion

Progress in the study of protective immunity to *T. parva* and development of an efficacious vaccine have been incremental and associated with the advancements in technology during the past 40 years (comprehensive review of early studies up to 1989 by Morrison et al. [27]). The major effectors of successful *T. parva* immunity are CD8^+^ CTLs specific for schizont-infected lymphocytes [1,11]. This protective CTL response is elicited both by natural infection and by the ITM [1]. Apart from the CD8^+^ CTL response, there are no other known immune correlates of protection in *T. parva*. A few studies have indicated a likely role for CD4^+^ helper T cells and γδ T cells [28,29,30,31], but assessment of the non-CD8^+^ T-cell response to *T. parva* has been limited. Furthermore, common measures of T-cell responsiveness, such as interferon gamma production and proliferation, provide no indication of protection from severe disease [12,13,14,15].

This substantial knowledge gap has significantly hindered progress on the development of an effective next-generation vaccine to prevent severe disease caused by *T. parva.* Several candidate antigens have been tested in cattle using multiple delivery platforms, but none have consistently elicited protective immunity in cattle [12,13,14,15]. To improve vaccine design and candidate vaccine assessment, more complete characterization of the successful immune response to *T. parva*, beyond cytotoxicity, is required. The goal of this study was to adapt a flow cytometry-based cytotoxicity assay used to study CTL responses to PRRS in pigs and *Mycobacterium* spp. in cattle [16,17] to the *T. parva* system to facilitate more complete assessment of responding T-cell populations following natural *T. parva* infection, ITM, or immunization. In contrast to the one-dimensional chromium-release assay, which was previously used to measure the CTL response to *T. parva* [11,13,14], the flow cytometry-based assay allows concurrent assessment of other cellular parameters, including surface markers, MHC–peptide antigen complexes, proliferation, and cytokine production, enabling broader characterization of cytotoxic effector cell populations, as well as responding T-cell populations that lack CTL activity.

The results of this study demonstrate that we have successfully adapted the flow cytometry-based assay for use with *T. parva*. Consistent with previous studies using chromium release assays [32], significant cytotoxicity was detected at all time points when *T. parva*-specific parent T-cell lines or CD8^+^ T-cell lines were used as effector cells. Unlike chromium release assays, this assay does not require use of radioactive material, and also allows quantitation of two different phases of cytotoxicity over time—early induction of apoptosis (annexin-V binding) and late increases in cell-membrane permeability (7-AAD binding) [33]. Since chromium release only occurs with increased membrane permeability, its measurements exclude early apoptotic cells, and are thus a less-sensitive indicator of cytotoxicity. In our studies, we were also able to ascertain additional basic phenotypic data on responding T-cell populations. The majority of PBMC from cattle stimulated with autologous, irradiated, *T. parva*-infected cells were positive for CD45R0 and negative for CCR7, consistent with an effector memory cell phenotype. Additionally, most of the cells expressed the T-cell activation marker CD25. In further studies, the presence of other cell surface markers, including markers of bovine γδ T cells, regulatory T cells, and various helper T-cell subsets, as well as the production of perforin, granzyme B, and different T-cell cytokines (e.g., IL-17, IFN-γ, and IL-10) could be explored in conjunction with cytotoxicity and proliferation, thereby providing a complete picture of the responding cell population.

One interesting finding of this study was a small, but significant, CD4^+^ CTL response to *T. parva*-infected cells. To the author’s knowledge, this is the first documented evidence of cytotoxic CD4^+^ T-cell activity against *T. parva*. Although observed less commonly than cytotoxic CD8^+^ T-cell responses, cytotoxic CD4^+^ T-cell responses have been documented in cattle infected with *Neospora caninum* [34,35] and in humans infected with *Toxoplasma gondii* [36,37]. Although highly unlikely due to the lymphocyte sorting and purification methods employed herein, in which we phenotyped CD4 and CD8 T-cell populations at multiple time points before and after separation using anti-bovine CD4 and anti-bovine CD8 mAB to verify purity of the CD4 and CD8 T-cell populations, it is conceivable that the small population of CD4^+^ CTLs could also exhibit low CD8+ expression and thus represent a “double-positive” T-cell population. This has been noted to occur naturally in healthy pigs, humans, and rodents, and in these species, T-cell activation tends to increase the percentage of double-positive T cells in circulation [38]. The phenomenon has not yet been noted in cattle [38]. The importance of cytotoxic CD4^+^ T cells in immune control of *T. parva* is unclear. Previous studies suggest that transfer of CD8^+^ CTLs, depleted of CD4^+^ T cells, between immune and naïve twin calves is sufficient to confer protection from *T. parva* [11]. However, whether CD4^+^ CTLs significantly augments protection in vivo remains to be demonstrated. In addition to more complete characterization of *T. parva*-specific CTLs, characterization of other components of the cell-mediated response necessary for CTL generation is crucial to successful vaccine development. The results of this study demonstrate that both CD4^+^ and CD8^+^ T cells proliferate in response to infected cells, and that activated, effector memory CD4^+^ T cells outnumbered CD8^+^ T cells. While few studies have touched on the potential role of CD4^+^ T-help in the development of a protective CTL response to *T. parva* [28,29,39], very little has been done to date to characterize necessary helper T-cell populations, or to elucidate how and when help is delivered.

A previous study conducted by Taracha et al. provided data showing development of CTLs against *T. parva*-infected cells only occurred in cultures containing *T. parva*-infected cells for antigen presentation to CD4^+^ and CD8^+^ T cells [39]. When CD4^+^ T cells were separated from the infected cells and CD8^+^ T cells with a transmembrane, development of functional CTLs was blocked. Similarly, ex vivo studies with *Mycobacterium a. paratuberculosis* (*Map*) have provided data that confirm the importance of the direct interaction of CD4^+^ and CD8^+^ T cells with antigen-presenting cells. In these studies, CTL responses were elicited against target *Map* antigens only when CD4^+^ and CD8^+^ T cells recognized their cognate antigenic epitopes at the same time [17,40,41]. This was demonstrated with the use of mAbs specific for MHC I and MHC II molecules. Antigen presentation to CD4^+^ and CD8^+^ T cells by antigen-pulsed dendritic cells was blocked in the presence of mAbs against MHC I alone or MHC II alone [41].

As shown in the present study, the methods developed for study of the immune response to a vaccine candidate peptide for *Map* can be used to study the immune response to *T. parva*. Using the assay described herein, similar in vitro and ex vivo studies can be undertaken in the *T. parva* system to fully characterize: (1) the phenotypic and functional characteristics of CD4^+^ helper T-cell subsets involved in the development of a successful CTL response to *T. parva;* (2) the kinetics of the interactions between CD4^+^ T cells, CD8^+^ T cells, and antigen-presenting cells that led to the development of a successful CTL response; and (3) how alterations in CD4^+^ T cell or antigen-presenting-cell populations (e.g., cellular phenotype and cytokine expression) affect the development of CTL responses to *T. parva.* The data generated in these studies will significantly assist researchers in vaccine development by guiding the selection of adjuvants and vaccine delivery platforms to point the immune response in the correct direction. Furthermore, the assays can subsequently be used to fully and objectively evaluate the immune response to *T. parva* candidate vaccines ex vivo.

In summary, we adapted flow cytometry-based methods used to study the CD8^+^ CTL response against *Mycobacterium* sp. and the PRRS virus to overcome a major technical impediment to the study of the immune response to *T. parva.* The ability to study both primary and recall CTL responses ex vivo allows for the dissection of antigen processing and presentation by APC, signals delivered at the time of Ag presentation, and the signaling and cross talk that occur between CD4^+^ and CD8^+^ T cells essential for generation of CTLs. Retention of the CD8^+^ CTLs before and after they provide the lethal hit of target cells will provide opportunity for further characterization of the phenotypic signature and factors regulating CTL function. Of importance for the study of *T. parva*, use of the methods has provided data that support earlier observations by Taracha et al. [39] regarding the likely importance of CD4^+^ T cell help in the development of a CD8^+^ CTL response to *T. parva*. We demonstrated that stimulation of PBMC from *T. parva*-immune cattle with autologous, infected cells elicits a proliferative response by both CD4^+^ and CD8^+^ T cells, and that those T cells exhibit phenotypic features consistent with activated effector memory cells. This assay can be used to elucidate immune correlates of protection and disease in *T. parva*, thereby facilitating the design of next generation vaccines to control this devastating livestock disease.

## Figures and Tables

**Table 1:** List of monoclonal and secondary antibodies used in the present study.

Monoclonal or Secondary Antibody	Antibody Isotype	Antibody Specificity	Antibody Supplier
ILA11A	IgG2a	Bovine CD4	WSUMAC, USA
7C2B	IgG2a	Bovine CD8	WSUMAC, USA
CACT80C	IgG1	Bovine CD8	WSUMAC, USA
ILA116A	IgG1	Bovine CD45R0	WSUMAC, USA
CACT116A	IgG1	Bovine CD25	WSUMAC, USA
GB21A	IgG2b	Bovine γδ TCR	WSUMAC, USA
3D12	IgG2a	Human CCR7	BD Pharmingen, USA
AKS8	IgG2a	Bovine CD335	Norwegian University of Life Sciences [23]
Goat anti-mouse	IgG2a PE.CY5.5		ThermoFisher, USA
Goat anti-mouse	IgG1 Alexa Fluor 647		ThermoFisher, USA
Goat anti-mouse	IgG1 FITC		ThermoFisher, USA
Goat anti-mouse	IgG3 FITC		Southern Biotech, USA
Goat anti-rat	IgG FITC		Southern Biotech, USA
Goat anti-mouse	IgG2b PE		Southern Biotech, USA

## Data Availability

Data presented in this study are available in the article.
